# Toward a neuromorphic microphone

**DOI:** 10.3389/fnins.2015.00398

**Published:** 2015-10-26

**Authors:** Leslie S. Smith

**Affiliations:** Computing Science and Mathematics, University of StirlingStirling, UK

**Keywords:** neuromorphic systems, sensory transduction, microphone, acoustic pre-processing, auditory system

## Abstract

Neuromorphic systems are used in variety of circumstances: as parts of sensory systems, for modeling parts of neural systems and for analog signal processing. In the sensory processing domain, neuromorphic systems can be considered in three parts: pre-transduction processing, transduction itself, and post-transduction processing. Neuromorphic systems include transducers for light, odors, and touch but so far neuromorphic applications in the sound domain have used standard microphones for transduction. We discuss why this is the case and describe what research has been done on neuromorphic approaches to transduction. We make a case for a change of direction toward systems where sound transduction itself has a neuromorphic component.

## 1. Introduction

Neuromorphic systems are electronic implementations of neural systems: originally in Mead (1989) the implementations were analog VLSI circuits, but more recently the term has come to be applied to digital VLSI and FPGA systems as well. Although the concept of neuromorphic systems is generally dated to Mead (1989), aspects of the ideas go back further (reviewed in Smith, 2010), particularly if one includes systems built from discrete components to emulate neural systems. Neuromorphic systems have been applied both to sensory systems, including the sensor itself (discussed further here), and to emulating parts of neural systems, from patches of neuron membrane to larger areas of neural tissue. Sensory system emulation has been one of the main elements of neuromorphic systems from the beginning primarily because it offers the possibility of real-time sensory processing, and this is critical in (e.g.,) autonomous robot applications.

Both real and synthetic sensory systems can be considered in three parts:

1. pre-processing: processing the signal arriving in the stimulus domain
2. transduction: performing the transduction of the signal flux (whether light, pressure waves, odor, or other) into a neural or electrical signal
3. post-processing: performing operations on this transduced signal.

**Figure 1:**
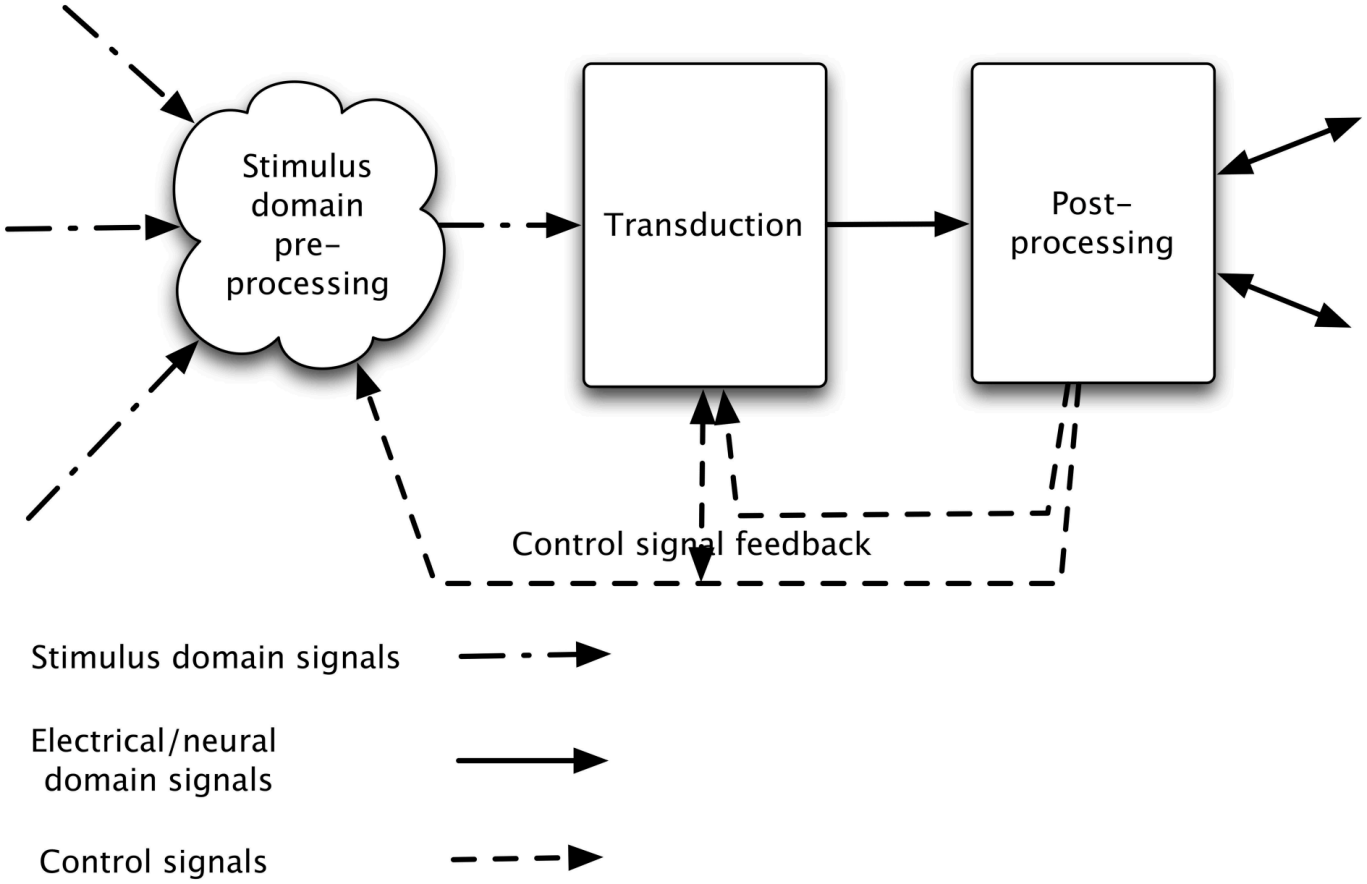
**Stages in a sensory processing system**. The incoming signal is processed in the sensory domain and this processed signal is transduced (into neural or electrical signals), and then passed to post-processing. In addition, general signal information (e.g., overall level) discovered at transduction is used to alter the stimulus domain pre-processing and aspects of the post-processed signal (e.g., which object or aspect of the signal is of interest) are used to alter both transduction and stimulus-domain pre-processing characteristics.

There is generally feedback of control information, adjusting the characteristics and operating point of the initial transducer, and possibly of the stimulus domain processing (see Figure 1).

What is it that makes a sensory system neuromorphic? Which stages above might be considered neuromorphic: pre-transduction signal conditioning, transduction itself, and/or post-transduction processing? To judge by the content of books and conferences on neuromorphic systems, it is primarily the second two: pre-transduction signal conditioning is often not considered neuromorphic. Yet if we are to consider synthetic sensory systems (going beyond the “electronic implementations of neural systems” definition above), effective pre-transduction processing is important.

We review briefly neuromorphic auditory sensory systems, considering them in the three parts identified above. Most neuromorphic research has been on visual systems and on processing sound signals *after* transduction, reviewed in Liu and Delbruck (2010). Work has also been done on neuromorphic olfactory transduction and post-processing (see Persaud et al., 2013), and there is also some work on tactile systems (reviewed in Saccomandi et al., 2014), and on echolocation (see Horiuchi, 2005).

Neuromorphic systems for many types of animal senses have been implemented, but although visual, olfactory, and to some extent tactile systems have included specifically developed transduction elements, auditory systems so far have almost always used standard microphones as the signal transducer (step 2 above). Most of the work on neuromorphic auditory transduction is on physical modeling of the cochlea and organ of Corti structures. Whether or not post-transduction processing is described as neuromorphic is generally a matter of the nature of the processing technique. If the processing is analog, or if it is digital hardware following a biologically inspired mode of processing, then it is often considered neuromorphic, whereas if it is purely software based, or if it is based on non-biologically inspired processing techniques, like Fourier transforms, it is not usually considered neuromorphic.

This review is organized as follows: Section 2 discusses auditory processing before transduction. Section 3 discusses animal auditory transduction and neuromorphically implemented versions of auditory transduction. Section 4 briefly discusses processing after transduction. Section 5 concludes by considering ways forward for neuromorphic microphones.

## 2. Processing prior to transduction

Both animal sensory systems and engineering implementations of sensory systems generally “process” the stimulus prior to transduction. This involves concentrating, altering, or focussing the signal (in the signal domain) in some way so that the greatest amount of useful information can be taken from the transducer(s) output by later processing. Animals often use this as part of active perception, hence the feedback loops in Figure 1. We briefly review signal domain pre-processing for the auditory modality, and then consider the similarities and difference across domains.

The auditory periphery of animals modifies the auditory pressure wave before it reaches the transducing system: in humans, the head, the ridges in the pinna, and the shape of the auditory canal differentially reflect and refract the signal in different parts of the spectrum on its way to the eardrum (tympanum) which is the beginning of the transducer itself. The precise characteristic depends on the direction of the sound source. This results in the so-called head-related transfer function (HRTF), characterizing the way in which the spectral distribution of a wideband signal is modulated by the relative location of the sound source to the tympanum. This aspect of the HRTF results in the same wideband sound having different energy distributions across the spectrum at the tympanum depending on the sound source location, and in particular making these generally different between the two ears. Animals also make gross ear, head, and body movements to alter the signal (differently) at each ear, and hence to assist them in locating the sound source they are interested in. Animals generally have two ears, and the relative difference in spectral energy is a fairly reliable cue for sound source location, partly because most sound signals of interest are wide-band, and partly because the head shadow will ensure that the HRTF is quite different for each ear. This provides the interaural intensity difference (IID) that is an important cue for sound direction finding.

In addition, where there is more than one sensor (and more than one pre-processing system), another cue is available. The time of arrival at each sensor will generally be different because the path lengths are different. This time difference [the Inter-aural Time Difference (ITD), sometimes called the Time Difference of Arrival (TDoA)] is generally between 10 and 600 μs (for humans), and although this is a very short time difference by biological standards, brains have adapted by creating some very rapidly responding circuitry (discussed in Graham et al., 2001). Echolocating animals take this one stage further and emit sounds, and then analyse the reflections to interpret their environment. Further, they dynamically alter both the sounds they make (see Surlykke and Moss, 2000), shortening or lengthening the pulse duration and the shape of the beam in which they are emitted (see Kounitsky et al., 2015) by altering mouth shape.

The middle ear can also modify the signal prior to transduction. The stapedius reflex rapidly reduces the intensity of particularly large sounds by stiffening the middle ear transmission chain. The primary purpose of this seems to be protection of the sensitive organ of Corti.

For engineering implementations, the purpose of pre-transduction processing may be to concentrate the sound from one direction at the transducer (hence enabling other sounds to be ignored), and/or to provide cues for the location of sound.

In the former case, multiple microphone arrays may be used to create a system which is sensitive to sound from one particular direction: this can be enhanced using reflectors as well (there is a good historical discussion of this in the acoustic radar section of the museum of retro technology website at http://www.douglas-self.com/MUSEUM/museum.htm, since this approach was used in tracking planes in wartime, with trained operators instead of transducers and post-processing).

In the latter case, pre-transduction processing (neuromorphic in the sense that it is modeled on animal hearing) usually means using a pair of microphones each possibly incorporating a reflector system so that both the inter-aural time difference (ITD) and across-spectrum inter-aural intensity difference (IID) provide good cues for sound location. Pu et al. (1997) use a pair of microphones with a reflector at each to emulate aspects of the HRTF and hence determine IID to find the direction of single speech utterances in quiet over a few 100 ms. Smith and Collins (2007) use a pair of omnidirectional microphones mounted on a flat panel to provide the ITD cue for sound source location. They also use post-transduction neuromorphic processing to enhance these cues. Given two microphones, the appropriate cues for sound source localization can be provided to a transducing system, although it is important to note that finding the ITD cues requires maintenance of the fine time structure of the sound. The cues might be enhanced by allowing the system to move the microphones and/or alter the reflector structure at each microphone, implementing active hearing and thus enabling rapid changes to be made to the pre-transduction system, so providing extra cues for sound location discovery. A very recent variant on this is to use a metamaterial based waveguide system that attenuates different parts of the spectrum in a direction sensitive way (Xie et al., 2015). For broadband sounds (like speech) this can be used to differentially amplify sound from one particular direction.

### 2.1. Similarities and differences from other sensory domains

In the auditory domain, the primary purpose of pre-transduction processing is to assist with sound source localization. There are similarities in the aims of the pre-processing techniques in other modalities because they also help address the *where* task.

The equivalent in the visual domain is the adjustment of eye or camera direction and of focus using a lens; in the olfactory domain the ability to sniff in different directions, and in the tactile domain, the movement of the sensors till they touch the object of interest. In all of these, the aim is to discover the location of entities, (whether sound sources, or visible or touchable objects, or odors), relative to the animal (or machine). These similarities reflect the importance of localization in different sensory domains. Further, in both animal vision and audition, there is machinery in place to attenuate the the incoming signal to avoid swamping the transducer itself. This is also the case in neuromorphic visual systems, but not so far in auditory systems, primarily because of the difficulties involved in adjusting the signal level prior to transduction.

There are however, major differences as well: there is no equivalent of focus in the olfactory domain, for example, and while turning the head (or the camera) entirely alters what is seen, the effect of turning the head on auditory signals is much more subtle, relating to alterations in the IID and ITD cues.

## 3. Transduction

We briefly review auditory transduction in mammals, and then discuss neuromorphic auditory transduction.

### 3.1. Auditory transduction in mammals

Biological transduction systems consist of structures with specialized transduction cells embedded in them. These cells turn what is being transduced (sound, light, or odorants, for example) into neural signals. They are often highly sophisticated, which is not surprising given that they are crucial for the organism's survival, and have been evolved over millennia to be as effective as possible. This is not the place for a full review of these structures: details of the visual, auditory, olfactory, and tactile systems, and the transduction cells can be found in appropriate textbooks (e.g., Part V of Kandel et al., 2012). What is common to them all, however, is that the transduction element is embedded in a cell that interacts with other cells, including nerve cells, to create a pattern of spikes or neuro-electrical signals.

Unlike microphones and cameras, neural auditory and visual transducers do not produce outputs which can easily be reconstituted into sounds or images. In the auditory system, the output (on the approximately 28,000 type 1 fibers of the auditory nerve) is a spiking signal. Each inner hair cell (IHC) transducer is innervated by 20–30 such fibers, so that the signal from each transducer results in up to 30 spike trains. The changes in the depolarization of the IHC result from the movement of the hairs (cilia). When each is displaced in one direction, ionic channels open allowing influx of K^+^ ions, and these channels close when the hair is displaced the other way. The type 1 auditory fibers code the level of depolarization at the hair cell: some fibers have a high spontaneous rate (firing rate in silence) and this increases and saturates at low signal levels, while others have lower spontaneous rates, but only saturate at much higher levels of sound. This enables the auditory nerve to cope with the large dynamic range of sounds that the environment produces. In addition, because of the nature of the cochlea as a series of high-pass filters the frequency response of each IHC transducer is asymmetric: it falls off rapidly as the frequency of the signal passes the most sensitive frequency of the IHC. In addition, the cochlea is active: the type 2 fibers of the auditory nerve innervate the outer hair cells, causing them to alter the damping of the membranes of the organ of Corti, resulting in a decrease of the Q (sharpness) of the frequency response of the IHCs as the signal level in that part of the spectrum increases. Because of the rectifying property of the depolarization of the hair cell, at lower frequencies (up to about 2 kHz in humans) the spikes on the type 1 fibers are in phase with the signal at the IHC. Thus, the output from the cochlear auditory transducer is about 28,000 spike trains, which together code the level of signal at each of the approximately 3000 IHCs. Together, they code the level of signal at each IHC, and, for lower frequencies, they also code the phase of the signal (Fuchs, 2010). At higher frequencies, they can code the envelope of the signal with about 2 ms precision (Dreyer and Delgutte, 2006).

Only aspects of the signal recoverable from the neural coding produced by the transducer are available for later processing. In that sense, what is produced by the transducer defines what is available in the neural system. Clearly, this requires appropriate sensitivity at transduction where the cells convert the original signal to neural signals. It is therefore unsurprising that very specialized transducer cells (and supporting cellular structures) are found in many animals, allowing further neural processing to achieve very great precision in sensory interpretation.

### 3.2. Neuromorphic auditory transduction

A variety of device types, often specialized transistors, have been used to convert sound, light, and odorant concentration into electrical signals: in addition, piezoelectric and capacitative effects have also been used in the tactile domain. Specialized phototransistors (plus some circuitry) have been used in the vision domain since (Mead and Mahowald, 1988). In the olfactory domain, specially coated transistors, which alter their conductance characteristics as the coating absorbs the odorant molecules or ions to which they are sensitive are often used. These may be ion-sensitive field effect transistors (ISFETs), described in Nakazato (2009). In the tactile domain, special-purpose mechanical transducers have been built, allowing emulation of both skin and vibrissae-based tactile systems. These have used a range of effects to create an electrical signal, from capacitance to piezoelectric to inductive and optoelectric effects, as described in Lucarotti et al. (2013).

Unlike the above sensory systems, neuromorphic auditory systems have not in general used a special transducer. Instead, they use a microphone to convert the audio pressure wave into an analog electrical signal. There are many different types of microphones, but their primary focus has always been accurate reproduction of the original pressure wave as an analog electrical signal, providing a single (analog) output. They are generally designed to have either a flat omnidirectional response (so that they are approximately equally sensitive to pressure waves at all frequencies, arriving from all angles), or to be essentially unidirectional (often with a cardioid response characteristic, though still with a flat frequency response), so that they pick up pressure waves primarily from a single direction. In that sense the (packaged consumer) microphone includes a small amount of auditory signal preprocessing.

But why is there so little work on neuromorphic auditory transducers? One reason is that microphone technology is very well-developed, reaching back to the late 1870's (see Berliner, 1891: for comparison, J. N. Shive discovered the phototransistor in 1948). There have been many developments since then, so that modern microphones are cheap, small, robust, and easily available. Another reason is that signal processing enables the creation of multiple signals from the initial microphone signal, with (for example) each signal containing only spectral content in some specific band. A very considerable body of work exists on taking the analog output from a microphone (generally amplified first), digitizing it, and passing it through various types of filter banks, (as discussed in Patterson et al., 1995, and reviewed in Lyon et al., 2010), and then coding it as a set of sequences of spikes, like the type 1 auditory nerve fiber output discussed in Section 3.1.

What advantages might there be in a truly neuromorphic microphone?

There are two particular capabilities that a truly neuromorphic microphone might have: the capability to perform active auditory sensing, and the capability to use multiple transducers sensitive to different parts of the spectrum. In active auditory sensing some of the transducer characteristics are changed (generally rapidly) because of changing auditory circumstances: for example, the sensitivity may be decreased when the sound level is high in some localized part of the spectrum. The use of multiple transducers (each sensitive to different parts of the auditory spectrum, either because each receives signals in a different part of the spectrum, as a result of acoustic pre-processing, or because each transducer is only sensitive to some part of the auditory spectrum) can also permit the reduction of masking of important quiet signals that occur near to (spectrally and/or temporally) louder signals. While it is true that such masking does occur in animals as well, it is still possible for animals to hear relatively quiet sounds in a noisy environment.

It can be argued that signal processing can achieve both of these capabilities. This however implies highly adaptive signal processing, rather than the fixed-program signal processing currently used. Further, where the overall level of the signal is very large, microphones may start to distort, whereas if multiple transducers with different spectral sensitivities (from, for example, acoustic bandpass filtering) are used, this distortion would be limited to the transducers in the spectral area where the signal is very large, decreasing the likelihood of saturation or overload, and allowing signals in other parts of the spectrum to be analyzed correctly. Modern digital signal processing is fast, but using acoustic pre-processing in conjunction with multiple transducers each with different spectral characteristics will have minimal latency. Further, given that each transducer is low power, reducing the amount of digital signal processing will reduce the power consumption of the system. Another advantage is parallelism: a multi-transducer neuromorphic microphone produces a parallel output, which is already divided up for processing in parallel. Where there are concurrent sounds (and this is the normal situation), recombination of (cues from) signals might be easier if there is parallelism in the sound representation right from the start.

For echolocating (sonar) systems, processing of the reflected signals needs to be very fast, so that the reduction in signal processing time due to spectral analysis in the transducer becomes more important.

**Figure 2:**
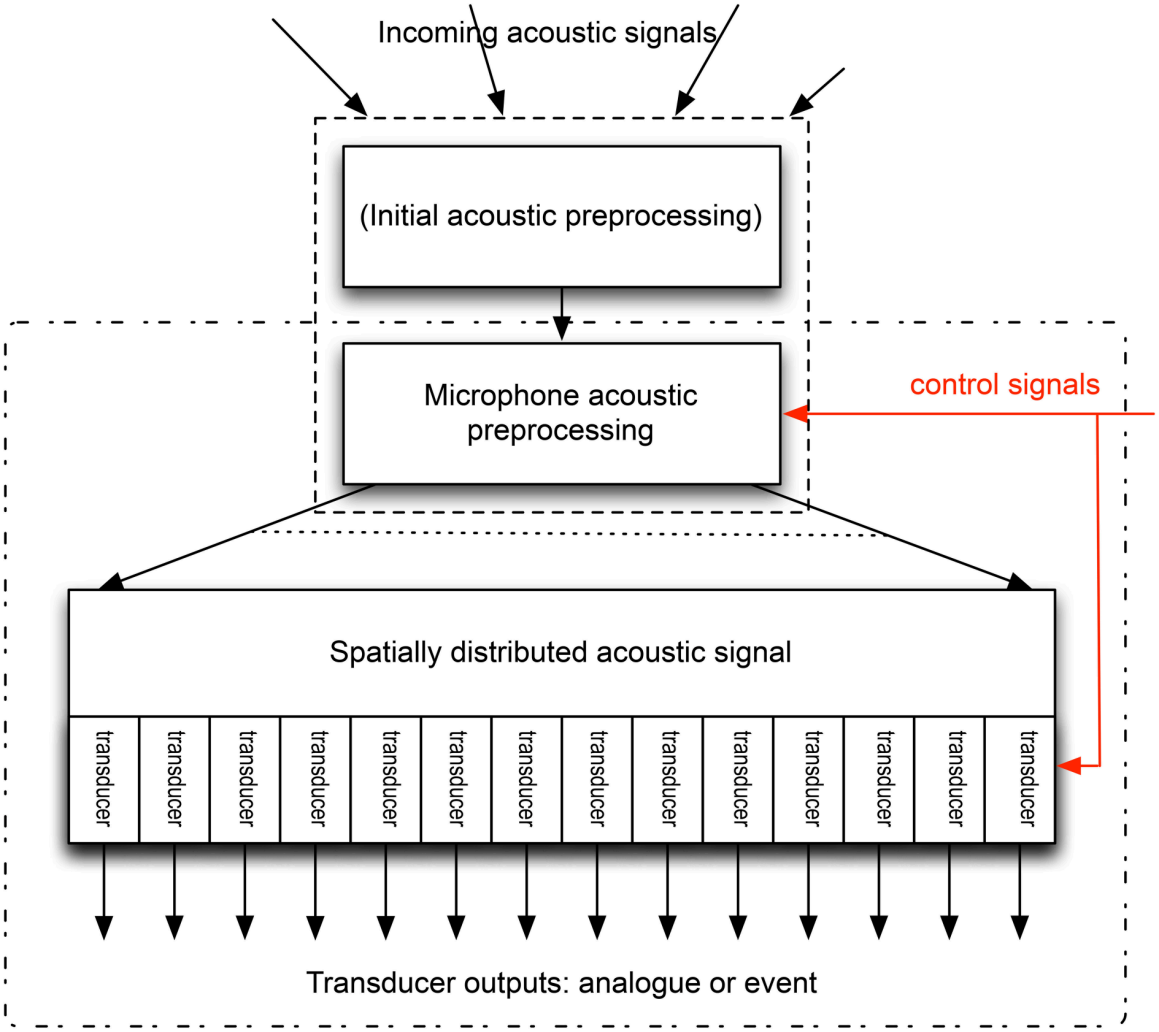
**Schematic of a possible neuromorphic microphone**. The incoming acoustic signal is altered pre-transduction: this may include the acoustic pre-processing discussed in Section 2, or the signal at the microphone may be the result of this processing, in which case the microphone consists only of the area enclosed by the dash-dot rectangle. The microphone acoustic pre-processing distributes the signal spatially, depending on the spectral content of the signal. This split may be continuous, or into a number of discrete signals. These are then transduced (by a number of transducers), whose output may be an analog electrical signal, or may be a code for this signal, such as an event (or spike) based code. The control signals (red) change the nature of the microphone acoustic processing, enabling rapid adaptation of this pre-processing, and can also alter the precise characteristics of the transducers.

What form might a neuromorphic microphone take? Figure 2 provides a schematic. If we are to use multiple transducers, then the acoustic processing must either translate the spectral distribution of the sound into a spatial distribution (this is what happens inside the cochlea, with the sensors being spread along the basilar membrane/tectorial membrane along the length of the cochlea), or each transducer must be responsive only to part of the spectrum. We can then place the transducers at appropriate locations, and produce an output from each, which might be an electrical signal following the pressure wave, or might be coded, as a sequence of events. But how might this be achieved in an engineering implementation?

### 3.3. Toward implementations of neuromorphic microphones

One strand of engineering implementations of neuromorphic microphones attempts to engineer a solution based directly on the cochlea and organ of Corti. Such physical cochlear modeling attempts to model the cochlea directly [as opposed to functional emulations, modeling the cochlea's effect, such as those of Patterson, Lyon, and Slaney (Slaney, 1988; Patterson et al., 1995), or computer modeling of the cochlear mechanics in detail, reviewed in Ni et al., 2014]. The earliest such work is by von Bekesy, (p. 522 et seq. of Von Bekesy, 1960) which uses pendula of different lengths suspended from a wire or rod. This model is much larger than the real cochlea, and was built to investigate the behavior of a multi-element resonator, rather than as a directly usable cochlear model.

Building a realistic cochlear-based model implementing a multi-transducer microphone is difficult for a number of reasons. Firstly, the cochlea relies on a fluid-filled substrate and a relatively poorly understood active mechanism for spectral filtering, and secondly it uses tiny mechanotransducers (the cilia of the IHCs) which work through the opening and closing of ion channels.

A very considerable amount of work has been done on detailed computational modeling of a fluid-filled cochlea. Much of this work has been concerned with fluid mechanics. For example including both inner and outer hair cells shows that an adaptive *Q*-value for the filters is possible (see Lim and Steele, 2002); Wang et al. (2014) show that such detailed modeling can provide accurate matching to experimental results. This work is aimed at better understanding of cochlear operation, rather than at creating a novel microphone.

There has been work on building such structures, with the history reviewed in Chen et al. (2006). Chen identifies one particular challenge as being building “an isotropic BM [basilar membrane] with stiffness similar to biological material”: silicon (and Si-compounds) are much stiffer than their biological counterparts. Another challenge identified is actually driving the system and observing its output. To achieve a low enough stiffness, Haronian and MacDonald (1999) use an array of micromachined beams (their patent covers both a MEMS based frequency separator, and an artificial cochlea), and (Tanaka et al., 1998) use a micromachined silicon fishbone-like structure which they drive acoustically and measure piezoelectrically. Wittbrodt et al. (2006) model the BM of the cochlea with a composite of polyimide and transverse ribs emulating the mechanical properties of the BM. Pressure waves are applied hydro-mechanically, and the movement is read using a laser vibrometer. White and Grosh (2005) test a silicon nitride (Si_3_N_4_) membrane, using vapor deposition: in 2006 (White and Grosh, 2006) built a BM from a stacked thin film structure using Si_3_N_4_/SiO_2_/Si_3_N_4_ with additional metallization (Au and Cr) to enable optical detection of movement. In 2008, White et al. (2008) report using the composite membranes with a fluid-filled chamber. They drive this at 100 dB acoustically, and measure vibration using an electrostatic/capacitative technique: this is a real microphone, but with very low sensitivity. Chen et al. (2006) builds an actual-size cochlea, with a metallized PVDF membrane. This is driven acoustically and measured with a laser vibrometer. They get good agreement on a number of measurements with the real cochlea. Again, the aim in these works is primarily understanding cochlea operation, rather than building a neuromorphic microphone, although (White et al., 2008) does produce a microphone which could be extended to have multiple transducers.

How might one produce a cochlear modeled microphone that actually has multiple transducers, sensitive to different parts of the spectrum, like the organ of Corti, but is practical to fabricate?

One possibility is based on an inverse harp (described as an “inverse piano” in Reichenbach et al., 2012): a harp has many strings, tuned chromatically over a number of octaves, plucked by the player to produce sound. However, in the inverse harp, instead of plucking the strings to produce sound, sound is played to the harp, and the energy content near the resonant frequency of each string causes that string to vibrate. This is then picked up by an array of magnetic pick-ups if the strings are ferromagnetic, (or could be optical if not). This would produce a multi-element sound transducer which can be driven directly, with the output captured directly. This would be inconveniently large, particularly for low frequencies, since these require long strings. Such a multi-element microphone has an additional problem, in that the strings will also respond to harmonics of their resonant frequencies. The BM in the organ of Corti is more sophisticated because the signal received at the different transducers has already had the higher frequency elements removed due to the nature of the traveling wave in the cochlea. This means that each resonator does not receive signals at higher frequencies resulting in a very sharp roll-off in sensitivity with frequency, as discussed in Section 3.1. It is as though the signal was applied initially at the shortest string, and what was not absorbed at that string was passed on to the next string. This provides an alternative form of neuromorphic microphone to the one in Figure 2, in that the acoustic pre-processing takes place at the string (resonator) rather than being the result of spatial distribution of energy from different parts of the spectrum.

What form should the output from such a multi-element transducer take? Standard microphones produce an analog signal accurately reflecting the pressure wave, as captured by the membrane in the microphone. Is it necessary to produce a large number of these in a multi-element microphone? Nature suggests not: the signal transferred by the (type 1 fibers of the) auditory nerve is a spike coding, as noted in Section 3.1. Signal strength is coded by the spiking rate over sets of these fibers: for each IHC, different fibers have different signal levels at which they start to fire and at which they fire at their maximal (saturating) rate, enabling each fiber to code part of a larger dynamic range.

Coding sound using an engineering approximation of this has been used in speech interpretation, originally in Ghitza (1986), and sound feature analysis for source location and musical instrument identification (see Smith and Fraser, 2004; Smith and Collins, 2007; Newton and Smith, 2012). In these systems, multiple spike trains are used for each section of the spectrum, with each spike coding the time of a positive-going zero-crossing of the signal. Spikes are generated only when the signal level in the half-cycle (calculated as $\frac{1}{2f_{s}}$ where *f*_*s*_ is the center frequency) exceeds a threshold, and the thresholds for the different spike trains are set as a geometric series, resulting in a logarithmic response. Although this might appear to lose information, there is good evidence that it is quite possible to resynthesize a good quality audio signal from this type of representation as shown in Pahar (2015). Ghitza (1986) found that this coding was effective in improving speech recognition, Smith and Fraser (2004) found it useful for onset detection, Smith and Collins (2007) found it effective for source location in noisy reverberant environments, and Newton and Smith (2012) used it for musical instrument classification.

Designing multi-element neuromorphic transducers that include spike based output has been attempted, primarily for cochlear implants. Kim et al. (2011) designed a finite element model of the cochlea and BM, modeling it in COMSOL, with a Si_3_N_4_ and polyimide BM model, and using a ZnO piezoelectric nanowire to produce output. Inaoka et al. (2011) developed a basilar-membrane like piezoelectric membrane which was implanted into a guinea pig cochlea. In addition to laser vibrometry used to measure displacement, piezoelectric output of up to 5 mV was generated in response to sound at 100 dB. Both of these studies are aimed at building an implantable device. Shintaku et al. (2013) have developed a BM system that they call a bionic auditory membrane, building on Inaoka et al. (2011). Though the eventual aim is piezoelectric signal generation, in this work, they have produced a 64 element array of beams from 4 to 142 μM in thickness. Laser vibrometry shows that these do respond to sound, though at 33 kHz for the 680 μM long beam.

Work done jointly by the author and the Integrated Micro and Nano System group at Edinburgh University attempted to combine MEMS and CMOS technology to produce a set of micromachined beams which acted as the gates of resonant gate transistors (RGFETs). Conceptually, beams would vibrate at a range of frequencies, and RGFETs would be placed to pick up this vibration and amplify it (see Koickal et al., 2011). It proved difficult to integrate the technologies although advances were made in the creation of long narrow beams (as discussed in Mastropaolo et al., 2012). It appeared that the stiffness of the beams, and tension in them after manufacture meant that driving the system acoustically to create measurable results proved impossible.

It is clear that considerable work still needs to be done to build a usable device, and this is discussed further in Section 5.

A different approach is to model a different type of ear. The ear of the parasitoid fly *Ormia ochracae* has been modeled, partly because it is so good at locating particular sounds [namely cricket stridulations (sounds)]. Ecologically, this is the sound it is particularly interested in. At least two different groups have developed MEMS based structures modeling this structure, which consist of two interconnected tympani, where the interconnection amplifies the difference in response. An et al. (2009) and Liu et al. (2013) use MEMS technology to model its two interconnected tympani, and use their model to understand the nature of the sensitivity of the fly system, building it to demonstrate the capabilities. Liu's group claim a 2° accuracy for a specific signal, namely an 8 kHz sine wave.

## 4. Post-transduction processing

A great deal of what animal brains do can be considered as post-processing of transduced sensory data. We briefly review what transduction in the auditory system (animal and synthetic) needs to be able to provide for this post-processing, and then consider the same question for neuromorphic systems.

### 4.1. Post-transduction processing for animal audition

All animal sensory systems use post-transduction processing: it is ecologically critical, and often extremely sophisticated, because it has to discover what is important for the animal from the signal. For audition, as for vision, this often means discovering, locating, identifying, and tracking objects, particularly those that might be prey, partners, or predators. As has been discovered by designers of artificial vision systems, this can be very difficult, particularly since vision has to cope with very variable illumination levels and shadows.

For auditory processing the nature of the processing (though not its sophistication) is different, because the cues for location and grouping of the energy that relates to a particular auditory source are very different, as discussed in Section 2, as is evidenced by the range and complexity of the nuclei of the auditory brainstem and mid-brain. These include specialized neural circuitry such as the giant synapses of the calyx of Held, and specific brainstem nuclei that appear to code ITD (the medial superior olive), and IID (the lateral superior olive) (Pickles, 2013). Unlike the situation in vision, it is the sound sources that need to be identified (and the reflections largely ignored, at least for the *where* task), rather than the light reflections that need to be identified in a way independent of the light sources.

Here, we are concerned with how auditory processing (or at least data modification) pre-transduction, and transduction techniques can provide a sufficiently rich signal for post-transduction processing. We are also interested in the feedback to the auditory periphery, since virtually all animal sensory systems include such feedback.

### 4.2. Post-transduction neuromorphic auditory processing

The aim of auditory neuromorphic post-processing depends on the application, but will include the “what and where” tasks: what is the sound source, and where is it? For locating sound sources, maintaining information about the precise timing of the signal is important. This is clearly the case for the ITD. For the IID, sub-millisecond precision is not important, but because of the rapid rate of change in wideband signals, comparison of spectral data between microphones still requires temporal accuracy. Sound source separation (in the sense of interpreting the sound source of interest, rather than solving the whole cocktail party problem) may also require information on the fine time structure of sounds, so that spectral elements with common onset, or common envelope, or common movement can be grouped together. It is therefore critical that neuromorphic auditory transduction maintains fine time structure. Auditory signals cover a wide dynamic range, and this means that the transducer also must be able to cope with a wide range of signal strengths.

There is a considerable amount of neuromorphic work on post-transduction processing, however, what is meant by post-transduction depends on the nature of the transducer output. When microphones are used for transduction, only a single signal is produced by each one and this follows the pressure wave at the microphone. As noted in Section 3.2, microphones often have a wide dynamic range (as this is crucial for their more common application in sound reproduction), and a flat frequency response. The signal available for post-processing therefore is more akin to that arriving at the eardrum of an animal, than the multiple sets of spike events actually sent to the brainstem along the auditory nerve. Thus, the initial post-transduction processing applied is generally multi-band bandpass filtering, modeling the pre-transduction processing carried out by the cochlea in animals, starting from Lazzaro and Mead (1989), and continuing through the more recent systems including van Schaik (1997), Liu et al. (2010), and reviewed in detail in Liu et al. (2014). Such systems can maintain the fine time-structure of the sound in each bandpass channel. This contrasts with the (generally software-based) computation of fast Fourier transforms and then mel-frequency cepstral coefficients (MFCC) by the speech recognition community who seem happy to discard the fine time-structure of the signal. This loses information critical both for source separation and for source location.

The cochlea is clearly more than a linear bandpass filter, and its active characteristics (for example, changes in response and alterations in filter selectivity with sound level) are included in the neuromorphic post-processing (of microphone based input) in the work of van Schaik's group (Fragnière et al., 1997; Hamilton et al., 2008). There is a detailed review of this in Liu et al. (2014). This work also maintains fine time structure, making its output more applicable to sound location finding, and sound streaming.

The animal cochlea (and presumably any multi-transducer system) also maintains fine time structure: in the case of the cochlea, the output from the transducers are events (i.e., action potentials on the auditory nerve type 1 fibers). Although it is clear that sound source direction is computed in the auditory brainstem in the lateral and superior medial olives, and it appears that attention to the foreground auditory signal is assisted, if not achieved, by the processing in the auditory brainstem and mid-brain (inferior colliculus in particular; Pickles, 2013), the precise mechanisms by which this is achieved are not known. Neuromorphic models of brainstem and midbrain processing therefore produce outputs that model the spectrotemporal receptive field properties produced by specific cell types, rather than signals that are known to be critical in the sound source location or separation problems.

Post-transduction processing that is also post-transduction in the animal system (and models brainstem or mid-brain outputs) is implemented neuromorphically in a number of papers: Fragniere and van Schaik detect amplitude modulation using a model of inferior colliculus mid-brain neurons (see van Schaik, 1997; van Schaik et al., 1998), and Glover et al. (1998) use a model based on the onset cells of the brainstem cochlear nucleus to detect onsets. van Schaik et al. (2010) note that such neuromorphic processing is essentially modeling the different characteristics of specific types of brainstem cells most frequently in the cochlear nucleus, which is where the type 1 fibers of the auditory nerve terminate. As a result, one is really modeling different conductances, and neuron shapes, as well as the network interconnecting them. Feeding back the results of post-transduction processing to alter the characteristics of the earlier components (pre-transduction processing, transducers, and filters) does not appear to have been implemented, although many neuromorphic filter banks adjust their selectivity depending on the signal level locally (Liu et al., 2014).

One can argue that the whole of the work on speech recognition using MFCCs is a form of post-transduction processing, but calling it neuromorphic seems inappropriate, because it is neither implemented in a neurophysiologically plausible way nor is its design directly influenced by biology.

## 5. Discussion

For sound, neuromorphic pre-processing (in the sense of reflectors etc. linearly altering the signal before the transducer), and neuromorphic post-processing (in the sense of circuitry that processes the signal after transduction), are already in existence for research auditory neuromorphic systems. The accessibility of cheap yet effective microphones has meant that the transduction stage has relied on these, unlike the transduction stages of other sensory neuromorphic systems. Recent developments in novel auditory transducers have been led by attempts to understand cochlear operation, and to create small low power systems that can be implanted to improve the effectiveness of cochlear implants. However, so far these have not found their way into research auditory neuromorphic systems.

Yet there are advantages to be gained by using neuromorphic microphones apart from understanding cochlear operation and biomedical engineering. Using a set of transducers that perform spectral separation directly removes the need for some of the post-transducer signal processing, resulting in a drop both in power consumption and latency. High-speed signal processing means that this latency is small (though there is a price in power to pay for this) but still important particularly when

- trying to alter the pre-transducer processing in the light of (e.g.,) sudden loud sounds interfering with speech
- trying to use an echolocating or echo based environment mapping system.

Using multiple transducers also means that one has the choice of attenuating or amplifying each of them independently (hence providing an extra mechanism for active auditory sensing), and of combining their outputs to emphasize or attenuate particular parts of the spectrum.

Which technologies might be best for neuromorphic auditory transducers is not yet clear. A technique that separates out the spectral content of the signal is required, but whether this should be a basilar-membrane like system, as used by Inaoka et al. (2011) and Shintaku et al. (2013), or as modeled by Kim et al. (2011) and Wang et al. (2014), or a set of independent resonators as suggested by Koickal et al. (2011) is not clear. Although MEMS technology can be combined with CMOS, adding liquids to this mix makes fabrication difficult, so that directly modeling the cochlea looks to be a difficult way forward. Some method for integrating the acoustic system that spreads out the spectrum over space and is integrated with a transducer array is required. At the same time it needs to have both sufficient sensitivity, and the capability for dealing with signals over a wide dynamic range.

As noted in Section 3.3, each transducer does not need to generate a precise rendering of the signal, but can provide an event or spike-based output capturing both phase (and hence fine time-structure) and signal amplitude, possibly using the technique described in Section 3.3. To generate a spiking output, one might either process the analog signals from each transducer, or else attempt to generate spikes directly using a piezo-electric element. Such phase-locked event (spike) based codes maintain fine time-structure without overhead. This can be particularly useful in sound streaming, where precise signal timing (see Brown and Wang, 2006) provides cues for sound streaming. Spike based outputs also produce less data, and multiple transducer outputs provide the data in a form that is ready for parallel processing.

There is currently considerable interest in new technologies for auditory processing, whether for wearable hearing aids, cochlear implants, or for use in autonomous robots. There are considerable differences in the requirements for hearing aids (of whatever sort) and for hearing for autonomous systems, because the former imply some form of re-synthesis of the signal, whereas the latter interpret the signal, without recourse to reconstructing it. However, where one wishes to re-synthesize only the foreground sound (or sound source of interest) in a hearing aid, rapid signal separation (an aspect of sound interpretation) is required, and this often entails using the fine time structure of the signal.

Biological inspiration need not be mammalian. Insects like *Ormia Ochracea* provide a very different model of hearing, although they tend to be specialized for specific tasks. In addition, novel materials that could provide better membranes for MEMS such as graphene are being investigated (see Choi et al., 2012; Grady et al., 2014), and these may provide more compliant and better conductive membranes, and hence become an interesting way forward.

### Conflict of interest statement

The author declares that the research was conducted in the absence of any commercial or financial relationships that could be construed as a potential conflict of interest.
